# Implementation of the mental health Gap Action Programme (mhGAP) within the Fijian Healthcare System: a mixed-methods evaluation

**DOI:** 10.1186/s13033-019-0301-z

**Published:** 2019-06-20

**Authors:** Fiona Charlson, Odille Chang, Ilisapeci Kubuabola, Jaclyn Schess, Catherine Latu, Ernest Hunter, Isimeli Tukana, Sefanaia Qaloewai, Rahul Shidhaye

**Affiliations:** 1 Queensland Centre for Mental Health Research, Brisbane, Australia; 2 School of Medical Sciences, CMNHS, Fiji National University, Suva, Fiji; 3 Pacific Research Center for the Prevention of Obesity and NCD (CPOND), Fiji National University, Suva, Fiji; 4 Yale University, New Haven, CT USA; 5 James Cook University, Cairns, Australia; 6 Fiji Ministry of Health and Medical Sciences, Suva, Fiji; 7 Public Health Foundation of India, New Delhi, India; 8 School of Public Health, University of Queensland, Brisbane, Australia

**Keywords:** Fiji, Implementation, mhGAP, Evaluation

## Abstract

**Background:**

To facilitate decentralisation and scale-up of mental health services, Fiji’s Ministry of Health and Medical Services committed to implementing the World Health Organization’s mental health Gap Action Programme (mhGAP). mhGAP training has been prolific; however, it remains unclear, beyond this, how successfully Fiji’s national mental health program has been implemented. We aim to evaluate Fiji’s mental health program to inform Fiji’s national mental health program and to develop an evidence-base for best practice.

**Methods:**

The study design was guided by the National Implementation Research Network and adhered to the Consolidated Framework for Implementation Research. CFIR constructs were selected to reflect the objectives of this study and were adapted where contextually necessary. A mixed-methods design utilised a series of instruments designed to collect data from healthworkers who had undertaken mhGAP training, senior management staff, health facilities and administrative data.

**Results:**

A total of 66 participants were included in this study. Positive findings include that mhGAP was considered valuable and easy to use, and that health workers who deliver mental health services had a reasonable level of knowledge and willingness to change. Identified weaknesses and opportunities for implementation and system strengthening included the need for improved planning and leadership.

**Conclusion:**

This evaluation has unpacked the various implementation processes associated with mhGAP and has simultaneously identified targets for change within the broader mental health system. Notably, the creation of an enabling context is crucial. If Fiji acts upon the findings of this evaluation, it has the opportunity to not only develop effective mental health services in Fiji but to be a role model for other countries in how to successfully implement mhGAP.

## Background

Reorganisation and decentralisation—ensuring access to treatments close to where people live and work—is crucial to scaling up mental health services [1]. Building on a the legacy of colonial centralisation (the 1978 Mental Treatment Act), Fiji’s Mental Health Act (2010) set the direction for deinstitutionalisation and provision of community mental health services [2], commencing with the establishment of Stress Management Wards in Divisional Hospitals and Mental Health Clinics at selected primary healthcare facilities. Further decentralisation is planned through the integration of mental health services into primary health care clinics, community mental health outreach services and psychosocial rehabilitation centres in each Division. This scale-up of mental health services will require a substantial increase in trained mental health workers, a strong plan for implementation of mental health strategies, and ongoing evaluation.

To facilitate these initiatives, Fiji’s Ministry of Health and Medical Services (MoHMS) committed to implementing the World Health Organization’s (WHO) mental health Gap Action Programme (mhGAP) as the backbone of its national mental health program. Launched in 2008, the WHO mhGAP aims to address the lack of care, especially in low- and middle-income countries, for people suffering from mental, neurological, and substance use (MNS) disorders. mhGAP provides health planners, policy-makers and donors with a set of clear and coherent activities and programmes for scaling up care for priority conditions. At the core of mhGAP is the mhGAP intervention guide (mhGAP-IG)—a tool for use by health-care providers working in non-specialised health-care settings. Fiji is one of the first Pacific Island nations to adopt mhGAP as its national mental health program and systematically train its workforce using this programme. Training of Fiji health workers in mhGAP was initiated by WHO and subsequently coordinated by the country’s Mental Health Unit. Training in how to use the mhGAP-IG has been extensive (approximately 678 health workers between 2014 and 2017); however, it remains unclear, beyond this, how successfully Fiji’s national mental health program has been implemented.

A recent systematic review identified 33 studies that have evaluated elements of mhGAP [3]; however, research has been predominantly focused on mhGAP training outcomes and, for the most part, has relied on pre-, post-test designs, which focus on changes in trainee knowledge, attitudes and clinical decision-making processes. A small number of other studies have assessed the outcomes of mhGAP in clinical practice, local adaptation of mhGAP, and economic modelling. The review identified the importance of reporting contextual strengths and challenges for implementation in the field; however, a comprehensive assessment of the implementation of mhGAP has not been undertaken anywhere in the world.

Across low- and middle-income countries, circumstances and resources have presented significant implementation challenges. Whilst a body of implementation research literature is beginning to emerge around mhGAP-centred service delivery [4, 5]; there is a dearth of evidence on mhGAP implementation processes and outcomes from which countries can draw upon to guide their own implementation. An assessment of the implementation of mhGAP in Fiji is a crucial step in assisting Fiji assess progress in its commitment to the decentralisation and scaling-up of mental health services. More broadly, the implementation of mhGAP in a multicultural, middle-income, island nation is considered with the view to lessons regarding implementation processes in other contexts with similar needs and challenges and contributing to a much-needed evidence-base for best practice.

We aim to evaluate Fiji’s mental health program by considering the three crucial components in achieving health outcomes proposed by the National Implementation Research Network proposes the combination of (NIRN) [6]—(1) effective interventions; (2) effective implementation, and; (3) an enabling context. NIRN proposes that if any component is weak then the intended outcomes will not be achieved, sustained, or used on a socially significant scale. We utilise a well-established implementation research framework which encompasses our knowledge gaps in relation to these components—the Consolidated Framework for Implementation Research (CFIR) [7].

## Methods

The evaluation design was guided by the Consolidated Framework for Implementation Research (CFIR)—a useful tool for organising and promoting synthesis of research findings, studies, and settings to stimulate theory development [7]. The CFIR has been used across a wide range of studies and provides a list of explicitly defined constructs for which data can be collected [8]. The CFIR explores 5 major domains: (1) intervention characteristics—the mhGAP programme; (2) outer setting—the external environment; (3) inner setting—the organisational context of health service setting; (4) characteristics of individuals, and; (5) the process of implementation. Framework constructs were selected to reflect the objectives of this study and were adapted where contextually necessary. Constructs included in the CFIR can be used to explicate elements defined within the Standards for QUality Improvement Reporting Excellence (SQUIRE) guidelines which are designed to promote knowledge-building for implementation and quality improvement studies by standardising how findings from these studies are reported. With this in mind, we structured the reporting of results in accordance with the domains and constructs of the CFIR.

We employed a mixed-methods (quantitative and qualitative components) process evaluation design utilising a series of instruments specifically designed to provide data for the domains and constructs of the CFIR. The first was a semi-structured questionnaire for completion by each participating health worker which captured data across the CFIR domains including their mhGAP training and supervision, application to practice and competency. Subsequent focus groups held at each study site further explored themes raised in the individual questionnaires and perspectives on the functioning of the broader mental health system and governance issues. A medicine audit was conducted at the facility level to asses availability of psychotropics medications.

Quantitative data from the individual questionnaires and medicine audit were extracted into Word excel and analysed using descriptive statistics in Stata 13.1 [9]. With participant consent, focus group discussions were audio-recorded and transcribed. Qualitative data from the individual questionnaires and focus group transcripts were analysed using a thematic framework drawn from the CFIR utilising NVivo 10 software [10].

Participants were selected from lists of health workers, provided by MoHMS, who had undertaken mhGAP training between the years 2014 and 2016. According to the training databases, a total of 678 staff had been trained in mhGAP at the time of this evaluation. To recruit maximum participant numbers within limited project resources we restricted our participants to mhGAP trained health workers currently working in primary health care facilities in the Central (6 out of 41 facilities) and Western (6 of 39 facilities) Divisions. Additionally, 10 key informants (KI) in senior management positions were interviewed within the health facilities; however, very few of these KIs reported having undergone the mhGAP training themselves. For each selected health facility, permission was sought from the divisional health sisters to visit and interview mhGAP trainees and written consent was obtained from all participants.

## Results

### Sample and participants

Of the list of 70 health workers identified for recruitment, 66 participants were available at the time of interviewing and included in this study (Table 1).

**Table 1:** Selected health facilities

Central Medical Division (n = 40)	Western Medical Division (n = 30)
1. Samabula Health Centre (n = 6)	7. Sigatoka Subdivisional Hospital (n = 3)
2. Nuffield Health Centre (n = 10)	8. Nadi Subdivisional Hospital (n = 7)
3. Makoi Health Centre (n = 5)	9. Lautoka Health Centre (n = 6)
4. Valelevu Health Centre (n = 6)	10. Ba Mission Hospital (n = 3)
5. Nausori Health Centre (n = 11)	11. Tavua Subdivisional Hospital (n = 5)
6. Navua Subdivisional Hospital (n = 2)	12. Rakiraki Subdivisional Hospital (n = 6)

Most participants were nurses, only 3 were medical officers. Over half were aged between 18 and 35, and almost 90% were female. Approximately two-thirds of our sample was posted at primary health care centres at the time of the study and approximately 60% of participants remained at the same position and stations as during their mhGAP training (Table 2).

**Table 2:** mhGAP-trained participant characteristics (N = 66)

Characteristic	Proportion of sample (%)
Participants age	
18–35	55
36–45	38
46–55	8
Gender	
Female	88
Male	12
Highest level of education attainment	
University UG	74
University PG	18
Blank	8
Current posting	
Health Centre	60
Subdivisional Hospital	32
Nursing Station	5
Others	3
Professional role	
Nurse	88
Medical officer	5
Counsellor	1
Subdivisional health sister	1
Other	5

### Intervention characteristics

#### Adaptability of mhGAP-IG

Feedback suggested that while mhGAP-IG is an appropriate and compatible tool for Fiji, there was no evidence of adaptation to the local context. It is unclear whether this was due to a perception that adaptation was unnecessary, or whether this process was yet to be undertaken.

#### Design quality and packaging of mhGAP-IG

Overall, participants had a positive view of mhGAP. In particular, participants found aspects of the mhGAP guidelines, like the flow charts, user friendly*: “we came to the health centre and I was talking amongst my friends and telling them how nice it is because it’s almost like the IMCI [Integrated Management of Childhood Illness] booklet […]. We don’t really have to remember all those things, we just carry the book.”*

### Outer setting

#### Patient needs

While some participants commented that current mental health services were viewed positively and accepted by patients and carers, with good outcomes—*“…she has noticed a big difference in the Dad. Now they are able to converse well”*—it was also noted that *“…we rarely receive [mental health] cases at our clinic”,* with informants identifying widespread stigma as a barrier to patients accessing mental health services*: “stigma remains in the community. People from the community don’t seek help because they don’t want to show their face. The family members are ashamed at having a mental case in their house.”*

Stigma reflects complex beliefs and behaviours and particular respondents identified the practice of accessing traditional and alternative healers as a barrier to people accessing mental health services. Regardless, interviewees reported that stigma reduction appears to have taken place at the health facility-level, facilitated by ongoing contact between general and mental health patients*: “stigma at the health facilities is becoming less. Before when we started the clinic way back in 2009, [patients from St. Giles Hospital attending the clinic] would sit away from other patients but now they are mingling and sitting together.”*

#### External policy

While several documents have been developed which relate to policies and the operationalisation of mhGAP in Fiji, they remain in draft form and have not been finalised or adopted by MoMHS. The recently drafted *National Mental Health and Suicide Prevention Strategic Plan (2016*–*2020)* has not yet been officially adopted or operationalised.

### Inner setting

#### Networks and communication within the mental health system

Overall, it was viewed that communication networks between different mental health services (e.g. St. Giles Hospital, public health, mental health units, and community health workers) were not functioning optimally with challenges noted in relation to referral pathways and information flow. While it was acknowledged that it was relatively easy to contact St Giles Hospital to discuss cases, around half of participants reported that successfully referring patients for specialist treatment was difficult. Further, the discharge of patients back into the community was experienced as problematic*: “it is the discharge bit that is not good [from St Giles to the community] because they […] are just given a card for the clinic day and no discharge summary. [It is not until] after 2 to 3* *months then the discharge happens. I think the referral pathway is OK, it’s just the discharge pathway.”*

Some suggestions for improving referral procedures were noted by the participants who drew from their experiences of other health programs, such as TB … *“for TB referral there is a standard form to fill in [and] if we had the same for mental health it would be easier.”*

In terms of referral procedures within a health facility (from nurses to Medical Officers in outpatient departments) 60% reported being aware of referral protocols which worked well. However, when asked to describe what those procedures were, there were large variations across individuals and facilities—descriptions typically related to the immediate step in the referral process. No reference to a standard protocol was made by any participant. Additionally, there were no apparent processes for tracking whether cases referred by outreach staff were being captured for follow-up at the health facility.

#### Readiness for implementation and implementation climate

Mental health was clearly a low priority within the health centres. When asked how participants prioritise activities within their daily work, 75% reported mental health activities as a moderate or low priority. When treating patients who come in for other conditions, screening for mental illness is infrequently considered, as noted by one informant: *“…we are just looking at maternal and child health and SOPD, and mental health will be the last one.”* Perceptions of the responsibility for this situation varied. Practitioners proposed that senior management needed to drive re-prioritisation “*if they were strictly told to use mhGAP, then they would have to do it. Otherwise they will do other things that they feel are more important.”* By contrast, informants in senior management roles identified a broader lack of priority for mental health by noting a lack of organisational commitment to the implementation mhGAP.

#### Leadership engagement and support

With few exceptions, feedback revealed a general lack of leadership in mental health. With one notable exception, across all sites no-one was allocated to support or facilitate the mhGAP implementation, that exception being a clinic with strong leadership, particularly from the mental health nurse, resulting in the largest number of mental health cases receiving treatment per month. The lack of leadership was compounded by poor engagement at higher service levels with very few doctors or management staff undertaking mhGAP training, Informants were clear that this should be addressed to encourage more collaborative patient care and avoid conflicting diagnoses and treatment strategies*: “there is a problem because they don’t understand the mhGAP*—*they have their own way of management”*—and—*“I think the supervisors should be the first one to go for that training. Only then will she strengthen the rest of us.”* Predictable flow-on consequences for support post-training were reported: *“I really understood the things so well. When I did the training I really wanted to implement it but then I had no support.”* Further, KIs also felt support from the mental health unit, which has a central coordination role for mental health services, was very limited.

#### Availability of resources

Limited availability of resources was identified as a substantial barrier to providing mental health services with transport frequently identified as a barrier to delivering home-based and outreach mental health services*: “we don’t spend much time with one patient because we have a lot to do with that one vehicle…”* Likewise, a lack of appropriate space and privacy for providing mental health services was noted as a barrier to providing appropriate care: “the *facility does not accommodate mental health patients here because there is not enough space and it does not have a room for a one to one counselling…That is why she is up in the hospital…”.*

Suboptimal mental health training of doctors was also noted as a significant issue, with nurses noting that, despite having the skills to assess patients and identify cases of mental illness, their abilities and potential roles are devalued by senior staff: *“…they send out Medical Officers who have not found their footing in mental health. So, we get frustrated when we refer patients to them. They should appreciate how valid the nurse assessment is.”*

However, the most commonly reported barrier in implementing mental health activities was time pressures experienced by health facility staff. Whilst over half of participants reported being available to provide mental health services during an average work day, two-thirds felt other responsibilities prevented them from doing so*: “I have to do clinic and then if the mental health nurse goes on leave today I have to do that also. I am doing four jobs in one day.”*

#### Availability and supply of psychotropic medications

Of mhGAP-trained participants, 57% felt that the availability of psychotropics was unreliable in their health facility. The medicine audits of each facility also revealed an inconsistent supply of medicines, with only selected antipsychotics and anticonvulsants being available continuously. No antidepressants were reliably available across all health facilities.

#### Clinical supervision and continuing education

**Fig. 1:**
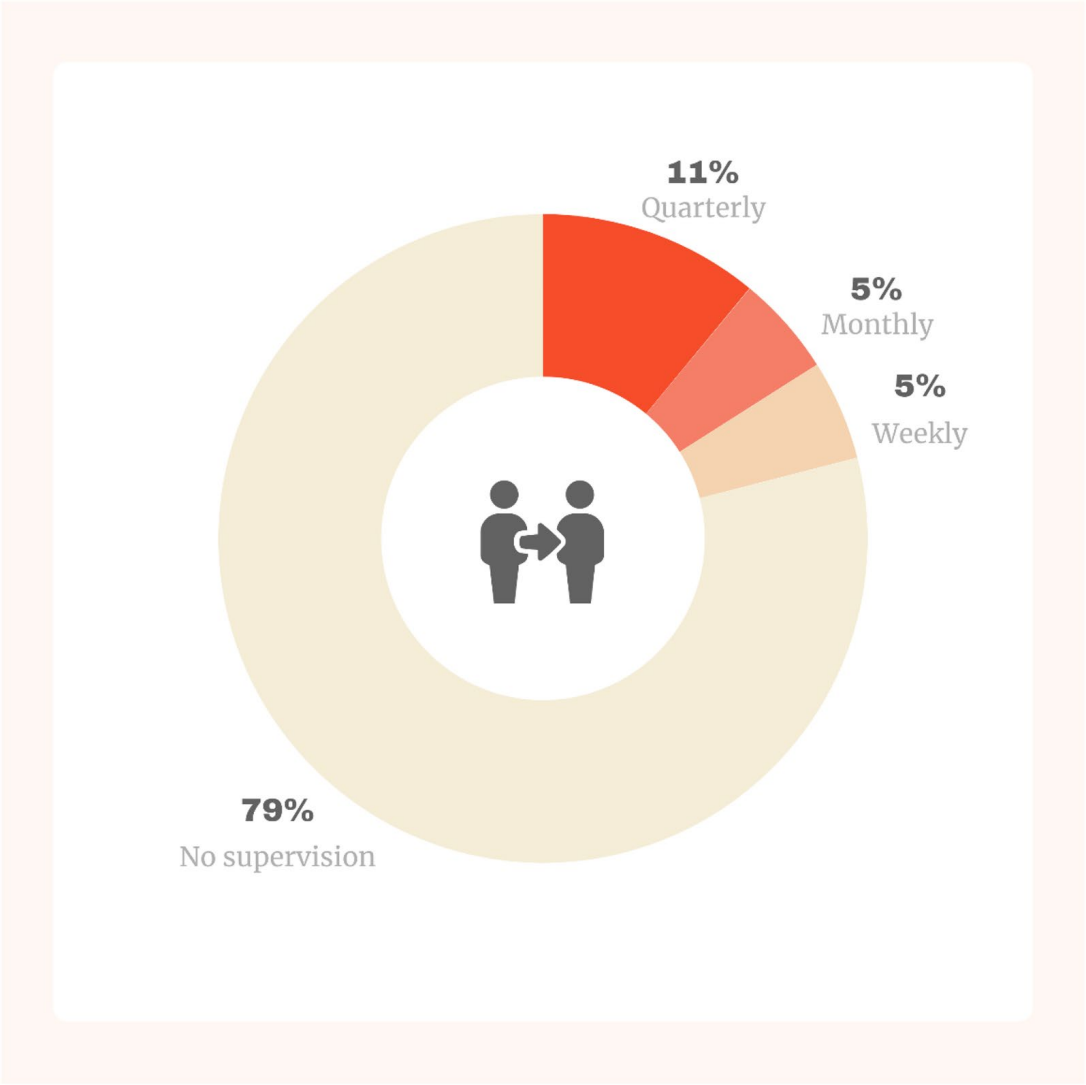
Frequency of clinical supervision

Of participants, 79% reported having received no clinical mental health supervision since their mhGAP training (Fig. 1). Among those who did report receiving some clinical supervision, it was most commonly provided by the subdivisional mental health nurse. The lack of supervision was noted as a substantial barrier to implementing mhGAP in practice. Participants unanimously felt regular supervision would greatly assist in conducting comprehensive patient assessments*: “we would like to be trained or accompanied by mental health nurses and for us to gain more experience ”.*

Roster scheduling also restricted health worker participation in clinical supervision opportunities.

*“The nurse usually comes with the teams for clinics, she asked around if we wanted to be with them and see how the clinic runs and after watching a few times we could do the clinics with the doctor. But we do shift work and sometimes we are not there, or we are on a day off, or we are covering in other places.”*

Of participants, 86% reported a desire to learn more about mental health: *1* *week of training is too short. We need to learn more so that we know how to provide a holistic approach to care and advocate for patients in an appropriate way.”* Participants noted major knowledge gaps remaining after mhGAP training and identified interest in: regular mhGAP refresher training; extension learning beyond mhGAP content; counselling skills; motivational interviewing skills; development of practical skills and experience, and; knowledge about medicines. The importance of practical experience was emphasised—for example, many participants reported having rarely or never seen patients with mental illness. It was felt this lack of experience and exposure to patients restricted their ability to put mhGAP into practice.

#### Access to information and knowledge

Discussion about access to information and knowledge was centred around the availability of the mhGAP intervention guide. Over 50% of respondents reported that mhGAP training materials were the only mental health resources available to them. Each participant reported receiving their own copy of the mhGAP-IG after training. However, facilities did not have any copies for common use and copies were frequently unavailable. This contrasted with other manuals which were provided, such as the IMCI guidelines.

When the idea was proposed to guarantee copies of mhGAP-IG inside examination rooms in the facilities, participants again drew upon experiences from other health programs and responded that this would increase their willingness to screen for mental disorders*: “…most of the time the IMCI nurses are not present but the IMCI guide is always present on our table. So, we are confident enough to just open the book and be guided through what to do.”*

### Characteristics of individuals

#### mhGAP training

Most participants attended only one mhGAP training. Nearly half of these trainings took place in 2015, with a further one-third attending training in 2016 or 2017. Around one-third of participants received training in all 11 modules of mhGAP. The most commonly received modules were for depression and psychosis, closely followed by bipolar disorders and epilepsy.

Nurses reported varying lengths of training, ranging from 2 days to 2 weeks. Those who reported at least 1 week training seemed satisfied overall, although some reported feeling that the course should have been longer. Those receiving only 2 days training consistently reported training time as inadequate.

Reports on the quality of the training also varied substantially. Most nurses were satisfied with the quality of the training and reported that the mhGAP training provided more mental health training than was provided in nursing school, with facilitators who were knowledgeable and experienced. However, some comments suggest opportunities for improvement; because of time constraints (sometimes due to late starts and poor organisation) training was rushed resulting in some participants not understanding challenging topics (e.g. psychosis, suicide and behavioural disorders) and not being given the opportunity for clarification. Other delivery issues included: ‘boring’ presentation styles/presenters; language barriers experienced with non-local trainers, and; a lack of hands on practice (when case scenarios, group work and the videos were provided the participants found this very useful).

#### Knowledge and beliefs about mhGAP

Clinical knowledge as assessed by the post-test for the mhGAP training demonstrated an average correct response rate of 67%. 22% of participants received a score of 80% or above; very few participants received a test score of less than 50%. Unfortunately, there is no pre-test data available to assess the change in knowledge as a result of mhGAP training; however, these findings are encouraging and suggest that, overall, knowledge about basic treatment of mental disorders is sound.

When asked about their perceived level of competency in using mhGAP, participants provided mixed responses with many reporting that they felt confident if they had a copy of the mhGAP-IG available to guide them during patient assessments. However, others reported that they had not had the opportunity to use mhGAP-IG in practice and no longer felt confident*: “we feel confident, but we need more practice. We wait for anyone who is referred from St. Giles so we can continue to practice…”*

#### Health worker’s stage of change

Participants who had undergone mhGAP training felt a strong shift in their views in relation to mental health. Many participants felt that the trainings raised their awareness around mental health and its treatment*: “I was never interested in [mental health] and when the sister in charge selected me to go and be part of the mhGAP training I told her I didn’t want to go. But I did go, and it was very interesting.”*

Most respondents felt more equipped in detecting and managing mentally ill patients and increased efforts to keep patients in community-based treatment (as opposed to inpatient)*: “[mhGAP] is very important because before, in the old curriculum, we were only concentrating on clients that had already been diagnosed [but] when you are trained with the mhGAP, you will be able to identify early.”*

There was also a positive change in views of, and attitudes towards patients, with participants reporting improvements in how they communicated with patients*: “our attitude, our behaviour towards them, our ability to listen to them all changed…”*

### Process

#### Planning and engaging

As noted previously, the planning of mhGAP implementation has been the responsibility of countries with little technical support and guidance from external agencies, unless specifically requested. Key implementation processes recommended by mhGAP include a national stakeholder’s meeting, needs assessment and identification of barriers to scaling-up. This should be followed by an action plan for scaling up, advocacy, human resources development and task shifting of human resources, financing and budgeting issues, information system development for the priority conditions, and monitoring and evaluation.

Those who attended mhGAP training reported that the process of implementation of mhGAP into their practices lacked planning and engagement, sentiments arising from the absence of discussions or communication from supervisors and senior management with respect to how mhGAP should be used within their health facility, and the lack of supervision and support received in integrating mhGAP into clinical practice*: “I came back from the training, nothing happened, we just continued with the normal routine that we used to.”* A lack of planning beyond the actual training was also noted by KIs: *“There are no plans of support beyond the actual training; no support, supervision or monitoring do before the mhGAP.”*

This experience contrasted with other training programs, such as IMCI and EPI, where the participants reported much clearer planning and engagement resulting in action and implementation of these programs after training*: “…we go for our IMCI training and when we come back, and we start doing IMCI. I’ll go for my pain ward training I will come back and do my colour coding and SOPD…”*

#### Executing

Participants reported very limited integration of mhGAP into practice. Many expressed that they have not had the opportunity to use their mhGAP knowledge to assess patients since the training. Mental health patients are primarily referred to and assessed by the doctors. It was reported that participants felt that there was little value in trying to assess patients when medical officers conduct their own assessment*: “sometimes we do not follow mhGAP because we are in triage and the medical officers do the proper assessment, so the guidelines are not really followed.”*

There was an overall perception that mental health is a vertical program that could not be integrated into their current work setting, especially in facilities where there is a mental health clinic. The importance of screening people for mental disorders does not seem to have been emphasised during mhGAP training.

**Fig. 2:**
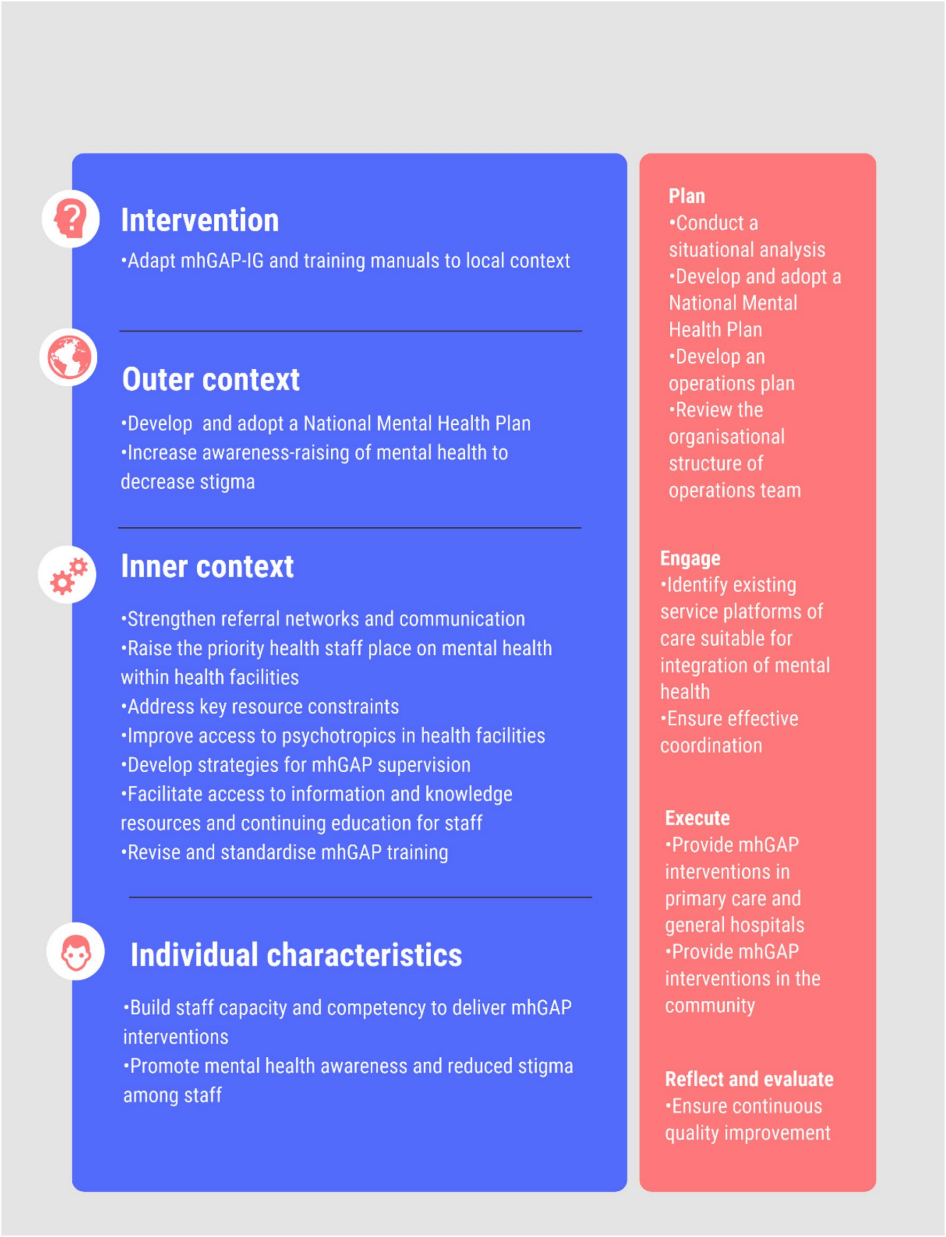
Summary of opportunities for mhGAP implementation

Raising awareness is a key activity of mhGAP. It increases demand for services from the community and reduces stigma. Of participants, only 40% reported ever participating in or organising activities to raise awareness of mental health issues in the community. Zone and community nurses have delivered awareness raising talks at village meetings as a part of outreach clinics; however, the extent to which this takes place appears limited*: “we don’t do actually awareness on mental health, but we go, and we take it as a package like it is part of non*-*communicable diseases.”* While there were reports of talks delivered to community groups such as churches and schools, these activities frequently aligned with annual events such as Drug Awareness Week, World Mental Health Day and World Suicide Prevention Day*: “nurses felt that creating more awareness in the community you result in more patients at the facilities.”* Furthermore, there was no mechanism to collect data on the execution of various care processes. The lack of monitoring meant that there was no opportunity for reflection or improvement of implementation processes. A summary of the opportunities for strengthening mhGAP implementation in Fiji are presented in Fig. 2.

## Discussion

Considering the three crucial components of NIRN [6]—(1) effective interventions; (2) effective implementation, and; (3) an enabling context—our study finds that Fiji has effective interventions within mhGAP which are considered valuable and easy to use. The implementation of mhGAP has seen some success, particularly with the health workers who had a reasonable level of knowledge and willingness to change how they deliver mental health services. However, there are substantial weaknesses and opportunities for change which can strengthen the Fijian health system and create a much needed enabling context.

Good governance forms the backbone of any health system. It comprises of accountability, responsiveness, open and transparent decision making and community engagement. Along with strong leadership provided by decision makers, key priorities in creating an enabling context for the Fiji mental health system to implement evidence-based interventions are to strengthen leadership and governance, which has been identified as a barrier to effective integration of mental health care in other low- and middle-income countries [11].

Evidence from different parts of the globe suggests that training of primary care physicians alone does not lead to improvement in detection and treatment of mental disorders [12, 13]. In addition to the training, it is important to address the structural, contextual and attitudinal barriers to motivate and support the primary care physicians (and other health care providers) to implement evidence-based interventions in a collaborative manner with the goal of achieving optimal patient outcomes and implementation fidelity. This can be achieved through provision of continuing implementation support. In Fiji, there is a key opportunity to create a ‘Technical Support Unit’ which can play the role of a facilitator and provide tools, training, technical assistance and support for quality improvement [14].

There exists significant opportunity for improvements within Fiji’s mental health services and a range of strategies for the implementation of health-care provider practices have been published elsewhere [15]. Specific evidence-based strategies which will address challenges identified in this evaluation exist and include; revising training practices to provide continuous in-service training and clinical supervision to staff (including ensuring competency in training and supervision practice), training of doctors and management staff in mhGAP, reorienting services to patient-centred and integrated care, capacity building of managers in change management to facilitate the implementation of integrated care, provision of anti-stigma workshops to staff and communities, strengthening communication networks, raising the priority of mental health, addressing key resource constraints by using existing resources more efficiently (e.g. redistribute resources from tertiary-level institutions to community-based services), establishing information and drug supply systems [11, 16].

Finally, the re-development and adoption of a national mental health strategy is crucial for Fiji. The processes for developing detailed and clearly documented Mental Health Care Plans have been successfully demonstrated by other countries such as India [17] and include synthesising the evidence on effective treatments for the target conditions, formative research that includes modelling the processes through which care will be delivered and pilot testing them before rolling out the intervention and a definitive evaluation of the final implementation is carried out. A Mental Health Care Plan for Fiji could ideally provide the details of care processes, platforms of care (facility and community), capacity building which includes training and implementation support and health systems strengthening activities.

### Evaluation limitations

It is important to note there were some keys limitations in this evaluation. One of the major limitations was our inability to use CFIR framework to design study tools. The post hoc use of CFIR may have resulted in missing out on some of the key constructs used in the CFIR framework. Another limitation was lack of patient involvement in program evaluation which in turn would have provided data on translation of provider knowledge into outcomes. Additionally, the representativeness of the participants included were restricted to current practitioners within the Central and Western divisions of Fiji. This was partially due to resource limitations and the need to prioritise geographies which had the most staff trained in mhGAP. For example; the Northern Division was excluded because in 2014–2015 only 10% of the total trained staff were from the Northern Division, in 2016–2017 there was no record of training in the Northern Division. It is unclear if that was because data was not recorded or there really was no training conducted for that year.

## Conclusions

This evaluation has unpacked the various implementation processes associated with mhGAP and has simultaneously identified targets for change within the broader mental health system. It has highlighted that whilst training of health workers is a necessary pre-requisite to translate evidence into practice, alone it is not enough to produce change. Implementation strategies need to be designed and operationalised, and health systems strengthened to address the barriers in implementation. If Fiji is able to consider and act upon the findings of this evaluation with a high level of commitment, it has the opportunity to not only develop effective mental health services in Fiji but to be a role model for other countries in the Pacific and beyond in how to successfully implement mhGAP within a strong mental health system.

## Data Availability

The datasets generated and/or analysed during the current study are not publicly available due reasons of participant confidentiality but are available from the corresponding author on reasonable request.

## Abbreviations

CFIR: Consolidated Framework for Implementation Research
EPI: Expanded Programme on Immunization
KI: key informant
mhGAP: mental health Gap Action Programme
mhGAP-IG: mhGAP intervention guide
MNS: mental, neurological, and substance use
MoHMS: Ministry of Health and Medical Services
NHMRC: National Health and Medical Research Council
NIRN: National Implementation Research Network
SQUIRE: Standards for QUality Improvement Reporting Excellence
WHO: World Health Organization
